# The copy number of the eukaryotic rRNA gene can be counted comprehensively

**DOI:** 10.1002/mbo3.1399

**Published:** 2024-03-04

**Authors:** Akinori Yabuki, Tatsuhiko Hoshino, Tamiko Nakamura, Keiko Mizuno

**Affiliations:** ^1^ Research Institute for Global Change Japan Agency for Marine‐Earth Science and Technology Yokosuka Kanagawa Japan; ^2^ Advanced Institute for Marine Ecosystem Change (WPI‐AIMEC) Yokosuka Kanagawa Japan; ^3^ Institute for Extra‐cutting‐edge Science and Technology Avant‐garde Research Japan Agency for Marine‐Earth Science and Technology Nankoku Kochi Japan

**Keywords:** biodiversity, diplonemids, ecology, eDNA, environmental microbiology, protists

## Abstract

Gene sequence has been widely used in molecular ecology. For instance, the ribosomal RNA (rRNA) gene has been widely used as a biological marker to understand microbial communities. The variety of the detected rRNA gene sequences reflects the diversity of the microorganisms existing in the analyzed sample. Their biomass can also be estimated by applying quantitative sequencing with information on rRNA gene copy numbers in genomes; however, information on rRNA gene copy numbers is still limited. Especially, the copy number in microbial eukaryotes is much less understood than that of prokaryotes, possibly because of the large and complex structure of eukaryotic genomes. In this study, we report an alternative approach that is more appropriate than the existing method of quantitative sequencing and demonstrate that the copy number of eukaryotic rRNA can be measured efficiently and comprehensively. By applying this approach widely, information on the eukaryotic rRNA copy number can be determined, and their community structures can be depicted and compared more efficiently.

## Graphical abstract

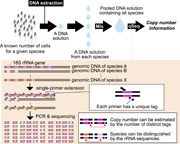

## 1 INTRODUCTION

The ribosomal RNA (rRNA) gene encodes a fundamental component of the ribosome, and its sequence information has been utilized in a very wide range of biological studies. For instance, the 18S rRNA gene (i.e., the eukaryotic small subunit rRNA gene) is most frequently used as a biological marker. The existence and distribution of microbial eukaryotes have been analyzed based on 18S rRNA gene sequences in environmental analyses (e.g., De Vargas et al., 2015). The 18S rRNA genes of many microbial eukaryotes have been sequenced and deposited in GenBank. Although the copy numbers of prokaryotic rRNA genes are well understood and summarized in *rrn*DB (Stoddard et al., 2015), it is much less known about their copy numbers in eukaryotic genomes. The copy number of the rRNA gene is important for addressing systematic bias when measuring community composition in molecular surveys based on rRNA gene abundance, and it helps provide a robust evaluation of the diversity and distribution of eukaryotes in the natural environment. Notably, the copy number has been counted in several protists using various approaches (Martin et al., 2022); some were estimated by quantitative PCRs (Zhu et al., 2005), and others were directly counted using genomic information (Maruyama, 2004). These approaches are sufficiently operational, but time‐ and effort‐consuming. Therefore, the establishment of a more efficient approach is highly expected.

Quantitative sequencing (qSeq) allows the simultaneous sequencing of amplicons and comprehensive gene quantification (Fu et al., 2011; Hoshino & Inagaki, 2017), and the unique sequence tags introduced by the single‐primer extension (SPE) at the first step at the end of a target DNA molecule are counted after sequencing to estimate the abundance of the target DNA. In the present study, we demonstrated that qSeq can be used to efficiently and comprehensively quantify eukaryotic rRNA genes.

## 2 RESULTS AND DISCUSSION

**Figure 1:**
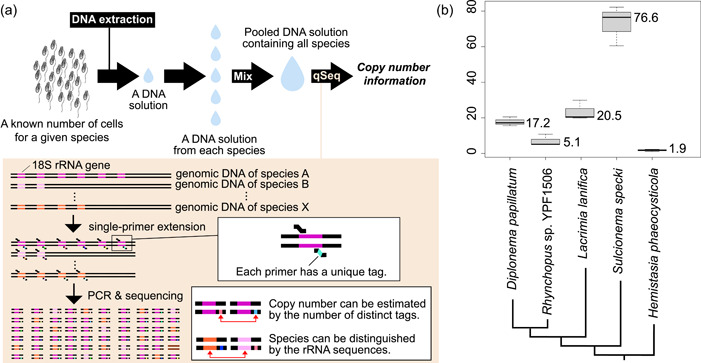
(a) Schematic image of the experimental process to comprehensively count the copy number of the eukaryotic rRNA gene. The more detailed process of qSeq was summarized in Hoshino and Inagaki (Hoshino & Inagaki, 2017). Drops represent DNA solutions. Arrows represent processes. The colored lines represent the rRNA gene, and different colors indicate different species. (b) The result of the rRNA gene copy number estimation of five diplonemids. The y‐axis shows the number of gene copies. The value next to the boxes is the median rRNA copy number of each species. The schematic phylogenetic relationships among the five species were referred from Tashyreva et al (Tashyreva et al., 2022). and the branch lengths do not represent their evolutionary distances. Small cells represent a known number of isolated cells for a given species.

We analyzed five species of diplonemids whose community structure and exchanges in natural environments have recently been focused on because this information may be useful for monitoring biodiversity in certain environments (Yabuki et al., 2021). The genomic DNA of each diplonemid species was extracted after counting the cells (Table 1). Simultaneous quantification and sequencing of diplonemid DNA were performed as previously described (Hoshino et al., 2021), with a few modifications. Briefly, after SPE) using S616F_Cerco primer (Fiore‐Donno et al., 2018) at 68°C for 10 min, 8 µL of ExoSAP‐IT Express (EIE) was added to 20 µL of SPE product to digest excess primers, followed by incubation at 37°C for 4 min. The SPE product treated with EIE was then used for the first round of PCR to specifically amplify diplonemid 18 S rRNA gene sequences. The primers used in first PCR were the S948_Dip primer (Yabuki et al., 2020) and an adapter primer that annealed to the region extended in the SPE reaction. The first PCR product was purified using agarose gel electrophoresis, followed by an index PCR for sequencing. After purification using AMpure XP beads, the indexed PCR products were sequenced using a MiSeq platform with the MiSeq Reagent Kit v3 for 600 cycles (Illumina). Quality trimming and merging of sequence reads were performed using Mothur ver.1.44 (Schloss et al., 2009). Unique DNA tags were counted as described previously (Hoshino & Hamada, 2017). The experimental process is summarized schematically in Figure 1a. More detailed information about materials and methods is summarized in Appendix A.

**Table 1:** Summary of the analyzed DNA and the copy number estimation.

Sample replicate	Cell number for DNA extraction	DNA concentration^a^ (ng/µL)	Abundance (copies/ng of DNA)	Copy number (copies/cell)		
				Each replicate	Average	Median
*Diplonema papillatum* ‐1	1 × 10^6^	1.22	1.3 × 10^3^	17.2	17.8	17.2
*Diplonema papillatum* ‐2	1 × 10^6^	0.94	1.5 × 10^3^	15.6		
*Diplonema papillatum* ‐3	1 × 10^6^	1.06	1.7 × 10^3^	20.5		
*Rhynchopus* sp. YPF1506 ‐1	3 × 10^5^	0.32	9.1 × 10^2^	10.8	7.0	5.1
*Rhynchopus* sp. YPF1506 ‐2	3 × 10^5^	0.21	6.5 × 10^2^	5.1		
*Rhynchopus* sp. YPF1506 ‐3	3 × 10^5^	0.23	5.8 × 10^2^	5.0		
*Lacrimia lanifica* ‐1	3 × 10^5^	0.21	2.6 × 10^3^	20.5	23.4	20.5
*Lacrimia lanifica* ‐2	3 × 10^5^	0.21	2.5 × 10^3^	19.9		
*Lacrimia lanifica* ‐3	3 × 10^5^	0.26	3.1 × 10^3^	29.9		
*Sulcionema specki* ‐1	1 × 10^5^	0.98	7.0 × 10^2^	76.6	73.2	76.6
*Sulcionema specki* ‐2	1 × 10^5^	1.21	6.1 × 10^2^	82.3		
*Sulcionema specki* ‐3	1 × 10^5^	0.73	7.4 × 10^2^	60.6		
*Hemistasia phaeocysticola* ‐1	3 × 10^5^	0.080	7.7 × 10^2^	2.3	1.8	1.9
*Hemistasia phaeocysticola* ‐2	3 × 10^5^	0.077	6.6 × 10^2^	1.9		
*Hemistasia phaeocysticola* ‐3	3 × 10^5^	0.072	4.2 × 10^2^	1.1		

^a^ The volume of DNA extract is 100 µL.

The obtained copy number of the 18S rRNA gene of each species from the three replicates was consistent (Table 1; Figure 1b); however, it differed among the species: the largest was approximately 77 (*Sulcionema specki*) and the smallest was approximately 2 (*Hemistasia phaeocysticola)*. As 20 copies of the 18S rRNA gene have been counted from the nuclear genome of *Diplonema papillatum* (Valach et al., 2023), our estimated score (i.e., approximately 17 in *D. papillatum*) is comparable to it and one‐sample t‐test also supported that there is no significant difference between two scores (t = −1.548, *p* = 0.262; Appendix). Hence, the estimation of the other four species can be expected to be reasonable. Ribosomal RNA genes exist forming tandem repeats in eukaryotic genomes and such repeated regions cannot be sequenced easily due to the technical problem. Therefore, it is still challenging to sequence complete eukaryotic genomes and count the rRNA gene copy number from them. The approach with qSeq can estimate the copy number, regardless of the complicated structure of eukaryotic genomes. Minor inconsistencies in the estimated copy numbers of *D. papillatum* may be due to technical errors in cell counting and/or DNA extraction. With qSeq, the efficiency of the 1st round of PCR does not affect the quantitative results. However, if the efficiency of the SPE reaction is not 100%, the copy number is underestimated. Further, if exonuclease treatment after SPE is insufficient, the primer may remain, leading to overestimation (Hoshino et al., 2021). As the cells of diplonemids are naked and their cells are easily lysed, we thought that their DNA was efficiently extracted; however, its efficiency is not 100% either and it may have also influenced the estimation partially. Nevertheless, the impact of possible artifacts in the experimental process was minor, and our estimation is reasonable. Although rRNA gene copy numbers have never been compared among diplonemids, this study revealed some variation. All diplonemids have similar lifestyles (i.e., planktonic flagellates feeding on microorganisms, including zooplankton and algae) and similar cell sizes; however, their rRNA gene expression may be regulated in different ways. Their copy numbers increased and/or decreased multiple times during evolution because they did not simply increase or decrease along with their phylogeny (Figure 1b). This variation is important when discussing the distribution and community structure of diplonemids. Species with higher copy numbers can be detected more easily using environmental DNA analysis, and the number of detected sequences does not simply correspond to their biomass. In the present study, we showed that qSeq can accurately quantify the rRNA gene copy number of five diplonemid species, indicating that it can be applied to other eukaryotic microorganisms for which the rRNA copy number is unknown. In particular, the ability of qSeq to simultaneously quantify the copy number of genes in many species is advantageous for analyses in natural environments. While the information about the copy number of barcode gene sequences from broad microorganisms increases, their biomass as well as biodiversity can be estimated at the same time by applying qSeq analysis with natural environmental DNA: the biomass can be estimated by analyzing the number of the barcode gene sequences in the template DNA sample with the information about the gene copy number per cell. For instance, the cell number of five diplonemid species analyzed in the present study can be estimated, when a given sample is analyzed with qSeq and the number of rRNA genes existing there is revealed. It is still unclear whether this analysis applies to microorganisms possessing rigid cell walls, such as some green algae and yeasts because their DNA cannot be occasionally extracted very well due to the cell walls. When targeting such microorganisms, a more careful approach to the lysis of their cells is needed. In addition to ecological aspects, the gene copy number information is also important in genetics and evolutionary biology. The difference in the copy number of a given gene within closely related organisms suggests that there is different adaptive evolution involved in the function of the gene. For instance, the variety of the rRNA gene copy number in budding yeasts is studied well and the adaptive advantages of possessing more and/or fewer gene copies have been discussed (Hori et al., 2023; Salim et al., 2017). As more efficient approaches to counting the gene copy number have also been expected in this field, our approach can be utilized for it.

## AUTHOR CONTRIBUTIONS

**Akinori Yabuki**: Conceptualization (lead); data curation (equal); formal analysis (equal); investigation (equal); methodology (equal); project administration (equal); resources (equal); supervision (equal); validation (equal); visualization (equal); writing—original draft (equal); writing—review and editing (equal). **Tatsuhiko Hoshino**: Conceptualization (equal); data curation (equal); formal analysis (equal); investigation (equal); methodology (equal); software (equal); supervision (equal); validation (equal); writing—original draft (equal); writing—review and editing (equal). **Tamiko Nakamura**: Investigation (equal); resources (equal); writing—review and editing (equal). **Keiko Mizuno**: Investigation (equal); writing—review and editing (equal).

## CONFLICT OF INTEREST STATEMENT

T.H. reported a potential conflict of interest related to patent royalties of the quantitative sequencing method for eDNA quantification. Other authors declare no competing interests.

## ETHICS STATEMENT

None required.

## Data Availability

The raw sequencing data for copy number estimation are available under GenBank BioProject accession number PRJDB16354: https://www.ncbi.nlm.nih.gov/sra/PRJDB16354.

## APPENDIX A ADDITIONAL INFORMATION ABOUT MATERIALS AND METHODS AND RESULTS

**Culture preparation and DNA extraction**

Five diplonemid species that have been kept as axenic cultures in our laboratory were utilized in the present analyses. Each species was grown in approximately 20 mL of Hemi medium at 20°C under the dark condition (Tashyreva et al., 2018), and the cells at mid‐exponential phase were subjected to be counted using a hemocytometer. Based on the cell density information, each culture was subdivided to contain a certain number of the cells shown in Table 1, and the tola DNA was extracted using the DNeasy Plant Mini Kit (Qiagen), according to the procedure provided by the manufacturer. Each DNA was eluted with 100 µL of elution buffer in total and the concentration was measured with Qubit (Table 1).

**Quantitative sequencing analysis**

Single‐primer extension (SPE) was conducted at 68°C for 10 min. The 20 µL SPE reaction mixture consisted of 1 × PrimeSTAR Max premix (Takara Bio Inc.), 5 µL of extracted DNA, and 300 nM of S616F_Cerco primer containing an eight‐base length random sequence tag (N8) and adapter sequence at 5′ end. The excess primers in SPE products were digested by adding 8 µL of ExoSAP‐IT Express (EIE) and incubated at 37°C for 4 min. After deactivating the enzymes, 5 µL of the SPE product was used for PCR with S948_Dip primer (Yabuki et al., 2020) and a primer complementary to the adapter sequence in the reverse primer in SPE. The 25 µL PCR product contained 1 × PrimeSTAR Max premix, 300 nM of each primer, and 5 µL of SPE product. The PCR involved 40 cycles of 98°C for 10 s, 55°C for 5 s, and 72°C for 5 s. The amplification product was subjected to agarose gel electrophoresis, and the band of the expected size was excised and purified using Nucleospin Gel and PCR Clean‐up Kit (Takara Bio Inc.). Finally, a second‐round PCR was performed to add an index for Illumina sequencing. The indexed PCR amplicon was purified using AMPure XP beads (Beckman Coulter), followed by sequencing using a MiSeq platform with MiSeq Reagent Kit v3 for 600 cycles (Illumina). All the qSeq reactions were triplicated for each diplonemid species. PhiX was not added in this study because N8 sequences help cluster identification at sequencing. The raw sequencing data for copy number estimation are available under GenBank BioProject accession number PRJDB16354.

**Data analysis**

Quality trimming and merging of sequence reads were performed using Mothur ver.1.44 (Schloss et al., 2009). Unique DNA tags were counted as described previously (Hoshino & Hamada, 2017). The experimental process is summarized schematically in Figure 1a. Briefly, quality‐trimmed and merged DNA sequences were assigned to 18S rRNA sequences of five cultured strains using Mothur. The N8 sequences at the 5′ end of the DNA reads assigned to each species were extracted and the number of unique N8 sequences were counted. In the counting, the number at the point where the number of the reads was 10 times the number of N8 was used for estimating the number of 18S rRNA genes based on Poisson distribution (Hoshino & Hamada, 2017). The estimated copy number of each species was plotted using boxplot in R 4.0.3 set in RStudio Version 1.3.1093.

**Statistical analysis**

One‐sample *t* test was conducted utilizing the following scores, which were estimated in the present and previous studies (Valach et al., 2023), and the results were summarized in Table A1. As the previous study (Valach et al., 2023) mentioned the possibility that *Diplonema papillatum* possesses more than 20 copies of rRNA genes, we also tested other possibilities; even if there are 21, 22, or 23 copies in the genome of *D. papillatum*, there is no significant difference with our estimation.

$$t=\frac{\bar{X}-\mu }{\frac{U}{\sqrt{n}}},$$

$$\bar{X}=17.767,U=6.243,n=3.$$

**Table A1:** Summary of the results of one‐sample *t* test.

*µ*	*t*	*p*
20	−1.548	0.262
21	−2.241	0.151
22	−2.934	0.099
23	−3.628	0.068
24	−4.321	0.0496
